# Pulmonary surfactant impacts in vitro activity of selected antifungal drugs against *Candida krusei* and *Candida albicans*

**DOI:** 10.1007/s10096-024-04799-7

**Published:** 2024-03-14

**Authors:** Alina Nussbaumer-Pröll, Peter Matzneller, Sabine Eberl, Markus Zeitlinger

**Affiliations:** Department of Clinical Pharmacology, Medical University of Vienna, Währinger Gürtel 18-20, 1090 Vienna, Austria

**Keywords:** Antifungal, Surfactant, In vitro, PD, MIC

## Abstract

**Purpose:**

This study investigates how surfactants affect the in-vitro anti-infective efficacy of micafungin, caspofungin, anidulafungin, and amphotericin B in treating pulmonary mycoses.

**Methods:**

MIC values for antifungal agents were determined against *Candida krusei* (now *Pichia kudriavzevii*) ATCC 6258, *Candida albicans* ATCC 90028, and 18 clinical isolates using the broth microdilution method in RPMI medium, following EUCAST recommendations. MIC assays included testing with and without Curosurf® surfactant at 1 mg/mL for *C. krusei* ATCC 6258 and all *C. krusei* isolates. Subsequent Time-kill studies in Sabouraud broth involved testing both *C. albicans* ATCC 90028 and C. *krusei* ATCC 6258 strains at concentrations equal their respective MIC values, with and without surfactant, using all four antifungals. CFU/mL were assessed at multiple time points up to 24 h. TKCs with different surfactant concentrations for *C. krusei* ATCC 6258 and mini-TKCs at various concentrations relative to the MIC of *C. krusei* isolates and the reference strain were conducted with micafungin, anidulafungin, and caspofungin.

**Results:**

MIC results showed that 1 µg/mL surfactant reduced killing of micafungin and anidulafungin against *C. krusei*, while caspofungin was unaffected. Amphotericin B's MIC decreased by half. TKCs demonstrated significant effects of surfactant on micafungin and anidulafungin against *C. krusei*, with complete abolition of anidulafungin's activity against *C. albicans*.

**Conclusion:**

This in-vitro study highlights the concentration-dependent inhibitory effect of surfactant on antifungal activity against *C. krusei* and, to some extent, *C. albicans*, necessitating further clinical validation for invasive lung mycoses treatment.

## Introduction

Pulmonary surfactant, a molecule consisting of a complex mixture of lipids and proteins, is a key component of epithelial lining fluid (ELF) and reduces surface tension in pulmonary alveoli. By coating the inner surface of the alveoli, it prevents alveolar collapse at low lung volume and additionally bears important biophysical and immunological functions [1]. Exogenous pulmonary surfactant has been investigated as a vehicle for improved antibiotic drug delivery to the lung [2–4]. Its advantageous properties are believed to allow for a more peripheral and uniform drug distribution, if compared with other methods of local drug delivery to the lung such as aerosol inhalation or direct intratracheal instillation [2].

Influence of pulmonary surfactant on the antimicrobial activity of selected antimicrobial drugs has been previously shown [5–7]. The significance of these studies lies in their findings, with daptomycin serving as a prominent example, as its antimicrobial activity was demonstrated to be inhibited in the presence of pulmonary surfactant [7]. The same is true for tobramycin, even if its inhibition by surfactant seems to be less marked [2]. In the case of daptomycin, this had heavy implications on the drug’s commercial success since it precluded its utilization for the clinical indication of community-acquired pneumonia.

In analogy, pulmonary surfactant might impact as well the anti-infective efficacy of antifungal drugs meant to treat pulmonary mycoses, as it has been reported in vitro by Rauwolf *et., al.* for aspergillus isolates [8]. Fungal pneumonia is a rare condition in immunocompetent patients, but is more frequent in patients with a compromised immune function. Patients with malignant haematological disease and patients under immunosuppressive treatment before, during or after hematopoietic stem cell or solid organ transplantation are particularly vulnerable to opportunistic, normally non-pathogenic fungi. Invasive pulmonary aspergillosis (IPA) accounts for the majority of cases, but invasive pulmonary mycoses caused by other orders of fungi including yeasts (e.g. *Candida spp.*), hyaline (e.g. *Fusarium spp.*) and dematiaceous fungi are reported as well [9, 10].

Our study aimed to examine how pulmonary surfactant (SUR) affects the antifungal activity of micafungin (MCF), caspofungin (CAS), anidulafungin (ANI), and amphotericin B (AMB) against *Candida krusei* and *Candida albicans*.

## Materials and methods

### Antifungals

Amphothericin B (Sigma, St.Louis, A4888).

Micafungin (“MYCAMIN” Astella Pharma).

Anidulafungin (powder, Originator PFIZER).

Caspofungin (powder, Originator Merck-Sharp & Dohme).

### Surfactant

Curosurf® (CUR), Chiesi Pharma.

### Growth media

RPMI-1640 2% Glucose, Sigma-Aldrich.

Sabaroud 2% Glucose Bouillon, Sigma-Aldrich.

Sabaroud 2% Glucose Agar, Sigma-Aldrich.

### Strains

Reference strains were obtained from the American Type Culture Collection (ATCC). Reference strains *C. albicans* ATCC 90028 and *C. krusei (now Pichia kudriavezevii)* ATCC 6258 were used. The 9 clinical isolates of *C. albicans,* as well as 9 clinical isolates of *C. krusei,* were collected from positive blood cultures and provided by the microbiological department of the General Hospital in Vienna.

### MIC

Minimal inhibitory concentrations (MIC) of amphotericin B (AMB), caspofungin (CAS), anidulafungin (ANI) and micafungin (MCF) were determined against *Candida krusei ATCC 6258 and Candida albicans ATCC 90028* as well as *n* = 9 clinical *C. krusei* isolates and *n* = 9 *C. albicans* isolates, respectively.

Additionally, MIC assays were conducted with and without the surfactant Curosurf® at a concentration of 1 mg/mL for *C. krusei* ATCC 6258 and all *C. krusei* isolates.

Broth microdilution was performed in RPMI growth medium following EUCAST recommendations (EUCAST E. Def 7.3.2 April 2020- Yeast Testing Directive Revised). In brief, test organisms were precultured overnight and were then introduced at an initial inoculum of approximately 5 × 10^5^ CFU/mL. Growth media contained defined concentrations of the respective antifungal in decreasing twofold steps. After 24 h of incubation at 37 °C, the MIC, defined as the lowest drug concentration giving rise to an inhibition of growth ≥ 50% of that of the drug-free control, was determined by using a microdilution plate reader measuring the absorbance at a wavelength of 450 nm.

### Time-kill studies and growth assesment

Antifungal killing of all tested antifungals was assessed in time-kill curves (TKC) against both *C. krusei ATCC 6258* and *C. albicans ATCC 90028*. In analogy, growth controls were done without antifungals in duplicate. All experiments were performed in Sabouraud-dextrose broth (SDB) alone and in the presence of SUR (Curosurf®) at a concentration of 1 mg/mL. For the compounds ANI and MCF, lower SUR concentrations (0.1 and 0.01 mg/mL, respectively) were tested against *C. krusei* ATCC 6528.

All time-kill curve (TKC) analyses and growth curve (GC) experiments were conducted for 24 h in a shaking water bath set to 37 °C with an amplitude of 22 mm and 150 amplitudes/minute under aerobic conditions. The fungal suspension was adjusted to 1.5 × 10^^8^ cells/mL in NaCl (0.9%), equivalent to a McFarland standard of 0.5, and then added to the test tubes to achieve a final concentration of 1.5 × 10^^6^ cells/mL.

At timepoint 0 (prior to antifungal addition) and at 1, 2, 3, 4, 6, 8 and 24 h, 100 μL samples were withdrawn and pipetted into the first row of a 96-well microtitre plate. Subsequently, seven serial dilutions with a volume of 20 μL each were performed in the remaining rows two to seven of the microtitre plates, with each well containing 180 μL of NaCl (0.9%). Aliquots of 20 μL from each dilution were plated onto Sabouraud agar plates and then incubated aerobically at 37 °C for 24 h. After incubation, colony-forming units (CFU) were counted, and CFU/mL values were calculated, considering the dilution steps. This calculation was performed using the formula: number of CFU multiplied by 5 × 10^n, where 'n' represents the dilution number.

### Mini- time kill experiments

In addition, killing of MCF, CAS and ANI against clinical isolates of *C. krusei* and ANI and MCF against *C. albicans* was assessed in mini-TKCs. To achieve this, fungal inocula were prepared following the aforementioned method of TKC experiments. They were then exposed to 4 times the MIC of the respective isolate of the compounds, both independently and in conjunction with SUR at concentrations of 1 mg/mL and 0.1 mg/mL, respectively. Colony counts were performed after 24 h of incubation. All experiments were performed in triplicate. For each pathogen, growth controls without addition of antifungal were performed in duplicate.

### Selection of antifungal concentration

For every different antifungal – yeast combination, selection of antifungal concentrations for time-kill experiments was based on previously determined MIC values. In general, the MIC itself as well as two multiples of the MIC (4- and 16-fold) were employed.

### Selection of surfactant concentration

Various surfactant concentrations ranging from 0.01, 0.1, to 1 mg/mL were chosen to assess whether even minimal doses could affect antifungal activity.

## Results

### MIC

Median MIC values with standard deviation for *C. albicans* ATCC 90028 and *C. albicans* isolates (Table 1) and for *C. krusei* ATCC 6258 *and C. krusei* isolates (Table 2), are shown*.* Isolates of *C. krusei* have been tested with and without 1 mg/mL surfactant. The median MIC in the presence of 1 mg/mL surfactant was at least twice as high as without surfactant for anidulafungin and micafungin e.g., isolate #1 1446 had a MIC with ANI of 0.06 µg/mL without SUR and rose to 0.125 µg/mL with SUR. For isolates #3 1448 and isolate #4 1449 ANI MICs increased up to 8- and 4- fold from 0.06 µg/mL to 0.5 and 0.25 µg/mL in the presence of SUR. While for amphotericin B, the median MIC was halved, caspofungin showed no discernible impact from the surfactant.

**Table 1:** Median MIC values with standard deviation (SD) in µg/mL for *C. albicans* ATCC 90028 and *C. albicans* isolates tested with anidulafungin (ANI), amphotericin B (AMB), micafungin (MCF) and caspofungin (CAS)

Strain	Median MIC of *C. albicans* isolates in µg/mL with SD							
	ANI	SD	AMB	SD	MCF	SD	CAS	SD
*C. albicans* ATCC 90028	0,03	0,02	0,06	0,04	0,0156	0,01	0,06	0,03
1446 #1	0,0156	0	0,06	0	0,0156	0	0,25	0
1447 #2	0,0156	0	0,06	0	0,0156	0	0,125	0
1448 #3	0,06	0	0,06	0	0,0156	0	0,25	0,19
1449 #4	0,06	0	0,06	0	0,0156	0	0,125	0,07
1452 #5	0,0156	0	0,06	0	0,0156	0	0,125	0
1453 #6	0,0156	0	0,06	0	0,0156	0	0,25	0,07
1454 #7	0,06	0	0,06	0	0,0156	0	0,125	0
1456 #8	0,03	0	0,06	0	0,0156	0	0,125	0
1458 #9	0,0156	0	0,06	0	0,0156	0	0,06	0,04

**Table 2:** Median MIC values with standard deviation (SD) in µg/mL for *C. krusei* ATCC 6258 and *C. krusei* isolates tested with and without 1 mg/mL surfactant (SUR) with anidulafungin (ANI), amphotericin B (AMB), micafungin (MCF) and caspofungin (CAS)

Strain	Median MIC of *C. krusei* isolates in µg/mL with and without SUR 1 µg/mL with SD							
	ANI	SD	AMB	SD	MCF	SD	CAS	SD
*C. krusei* ATCC 6258	0,03	0,02	0,25	0	0,06	0,04	0,25	0,07
*C. krusei* ATCC 6258 + SUR	0,06	0	0,125	0,00	0,125	0	0,25	0
1261 #1	0,06	0	1	0,29	0,125	0	0,25	0
1261 #1 + SUR	0,125	0	0,5	0	0,5	0	0,25	0
1303 #2	0,06	0,04	1	0	0,125	0,07	0,25	0,14
1303 #2 + SUR	0,06	0,00	0,5	0	0,25	0	0,25	0
1351 #3	0,06	0	0,75	0,71	0,125	0	0,25	0
1351 #3 + SUR	0,5	0	0,5	0,00	0,5	0	0,25	0
1410 #4	0,06	0	1	0	0,25	0	0,5	0,14
1410 #4 + SUR	0,25	0	0,5	0	0,5	0	0,25	0
1420 #5	0,06	0	1	1	0,125	0,07	0,25	0,14
1420 #5 + SUR	0,125	0	0,5	0	0,25	0	0,25	0
1431 #6	0,125	0,04	0,75	0,29	0,125	0	0,25	0
1431 #6 + SUR	0,125	0,00	0,5	0,00	0,25	0	0,25	0
1432 #7	0,125	0,04	1	1	0,25	0,07	0,25	0
1432 #7 + SUR	0,25	0,00	0,5	0	0,5	0	0,25	0
1441 #8	0,125	0,04	1	0	0,125	0	0,25	0
1441 #8 + SUR	0,125	0,00	0,5	0	0,125	0	0,25	0
1450 #9	0,06	0	1	0	0,125	0	0,5	0
1450 #9 + SUR	0,25	0	0,5	0	0,25	0	0,5	0

### Growth curves

**Fig. 1:**
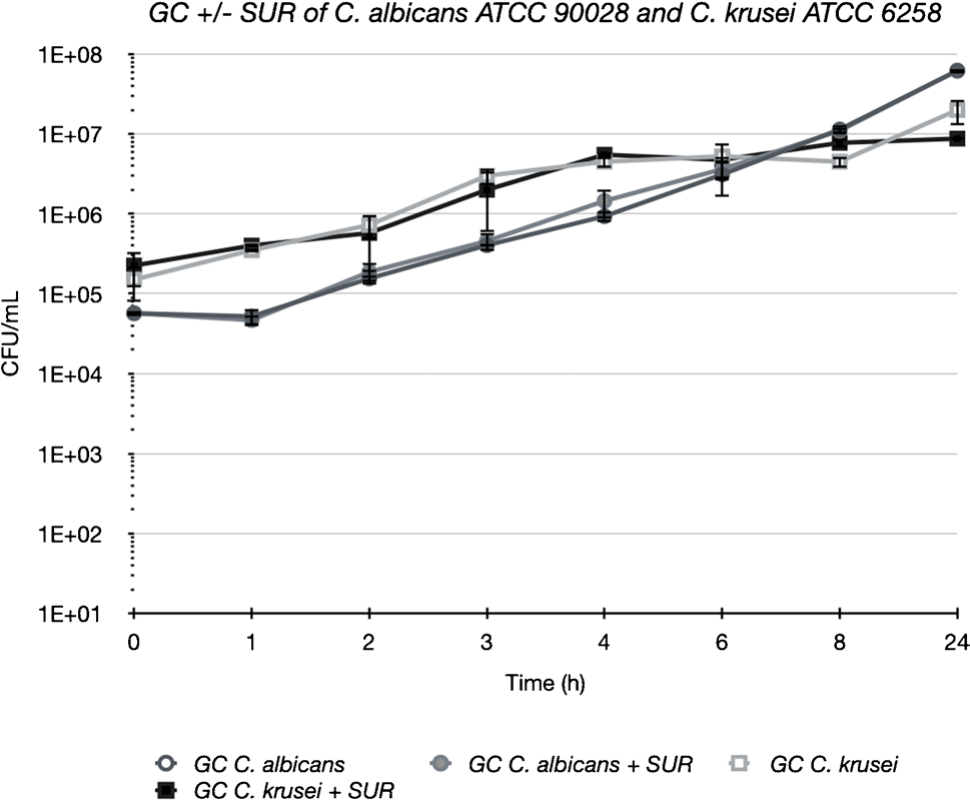
Growth curves of ATCC strains of *C. albicans* and *C. krusei* with (closed symbols) and without (open symbols) 1 mg/mL surfactant

Growth curves of ATCC strains were not influenced by addition of surfactant and reached after 24 h ~ 1 × 10^^7^ CFU/mL with *C. albicans* and ~ 1 × 10^^8^ CFU/mL with *C. krusei* (Fig. 1).

### Time kill curves

**Fig. 2:**
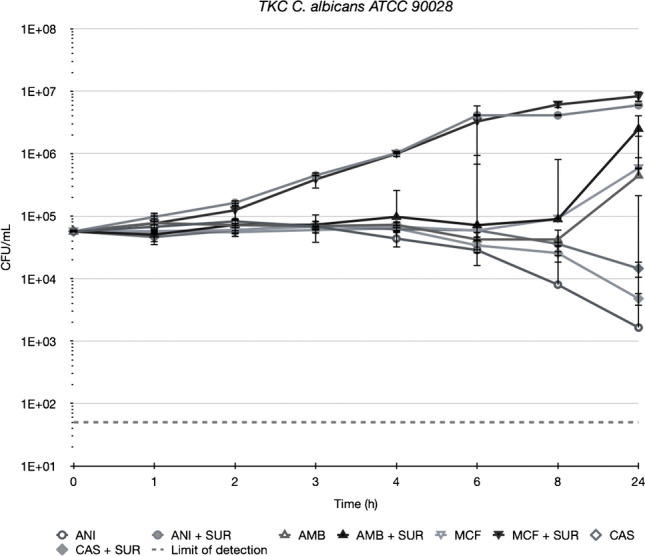
Killing of anidulafungin (ANI), amphotericin B (AMB), micafungin (MCF) and caspofungin (CAS) against *C. albicans* ATCC 90028 in Sabouraud-dextrose broth (SDB) alone (open symbols) and in the presence of 1 mg/mL of pulmonary surfactant (SUR, closed symbols). All compounds were tested at a concentration equal to their respective MIC against the test strain

Time Kill Curves conducted with *C. albicans* ATCC 90028 indicated that the addition of 1 µg/mL of SUR reduced the efficacy of ANI, MCF, and CAS all tested at a concentration of 1xMIC (Fig. 2). This effect was most pronounced for ANI (open circle), where the CFU/mL decreased to 1 × 10^^3^ CFU/mL without surfactant, compared to 1 × 10^^7^ CFU/mL with ANI + SUR (filled circles). Similar trends were observed with MCF and CAS (triangles and squares).

**Fig. 3:**
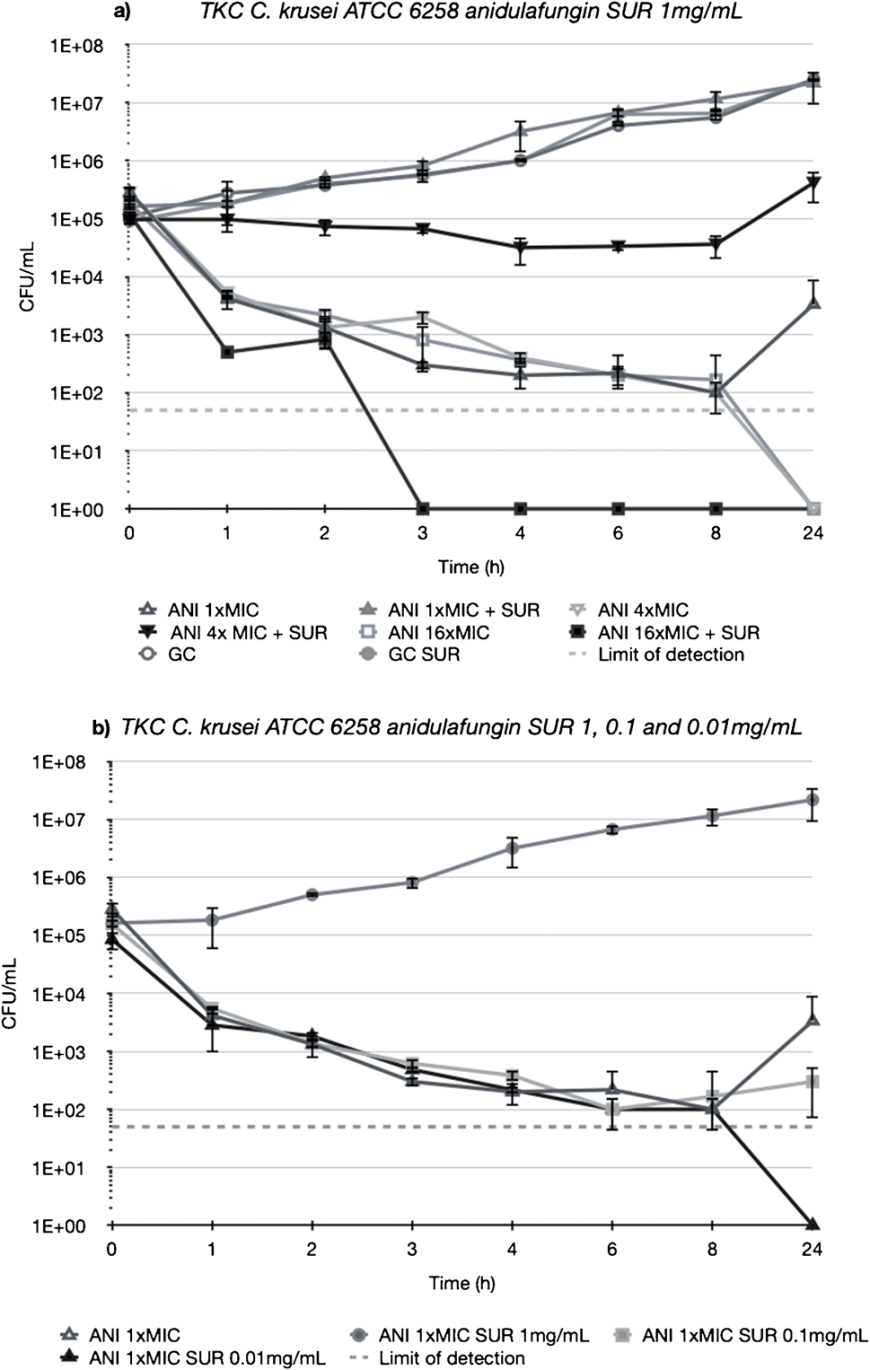
(**a**) Killing of different concentrations of anidulafungin (ANI) against *C. krusei* ATCC 6258 in Sabouraud-dextrose broth (SDB) alone (open symbols) and in the presence of 1 mg/mL of pulmonary surfactant (SUR, closed symbols). ANI was tested at 1, 4 and 16 times its MIC against the test strain. (**b**) Killing of ANI 1 time its MIC against *C. krusei* ATCC 6258 in SDB alone (open triangle) and SDB enriched with different SUR concentrations of 1, 0.1 and 0.01 mg/mL (filled symbols)

**Fig. 4:**
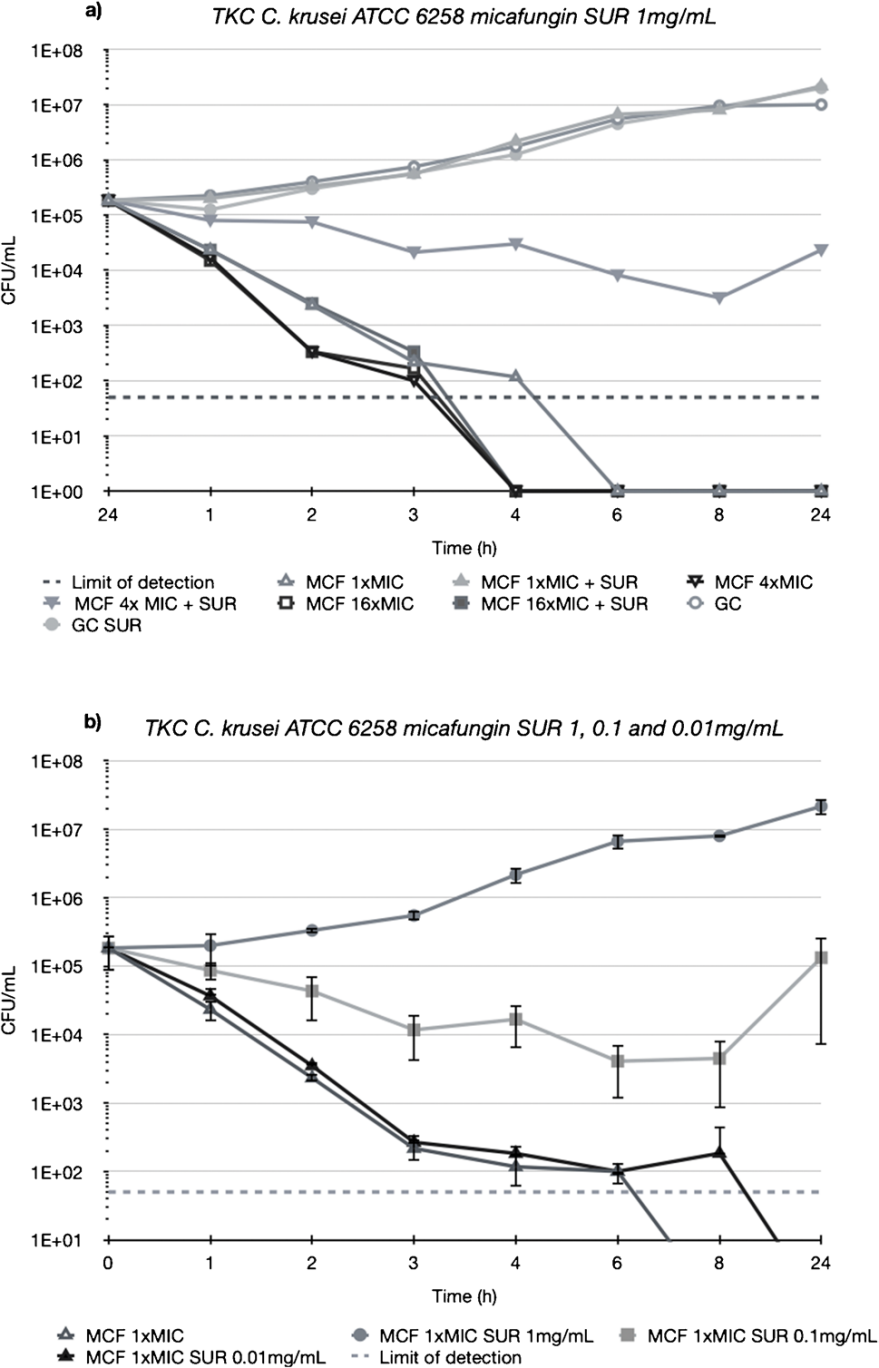
(**a**) Killing of different concentrations of micafungin (MCF) against *C. krusei* ATCC 6258 in Sabouraud-dextrose broth (SDB) alone (open symbols) and in the presence of 1 mg/mL of pulmonary surfactant (SUR, closed symbols). MCF was tested at 1, 4 and 16 times its MIC against the test strain. (**b**) Killing of MCF 1 time its MIC against *C. krusei* ATCC 6258 in SDB alone (open triangles) and SDB enriched with different SUR concentrations of 1, 0.1 and 0.01 mg/mL (closed symbols)

**Fig. 5:**
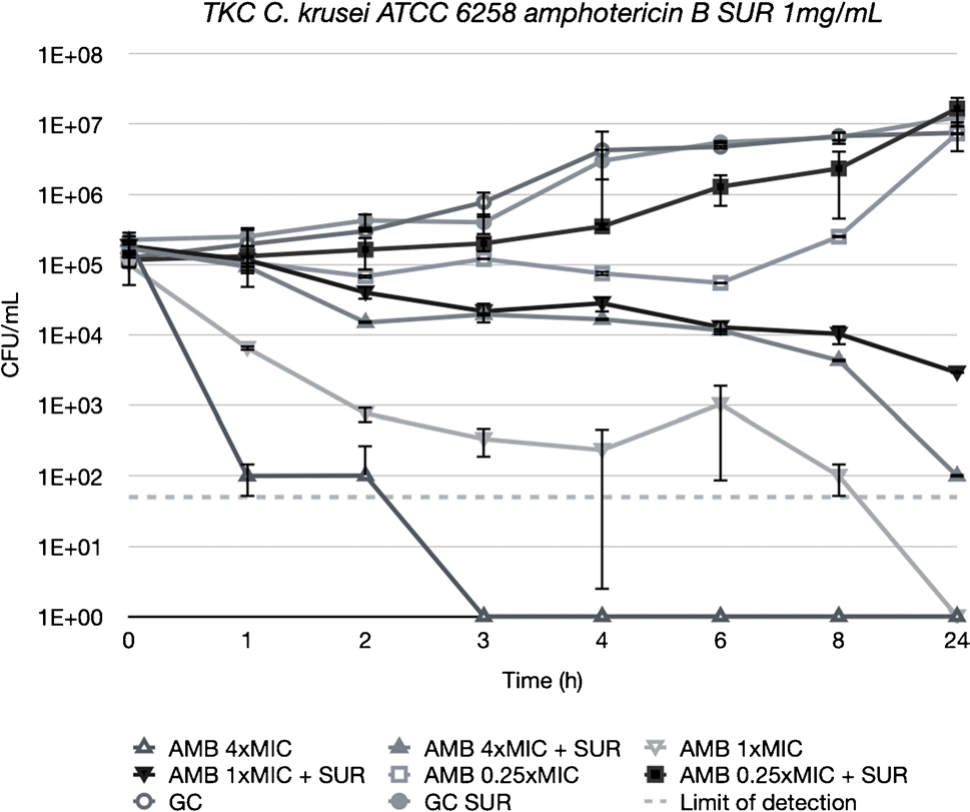
Killing of different concentrations of amphotericin B (AMB) against *C. krusei* ATCC 6258 in SDB alone (open symbols) and in the presence of 1 mg/mL of SUR (closed symbols). AMB was tested at 0.25, 1 and 4 times its MIC against the test strain

**Fig. 6:**
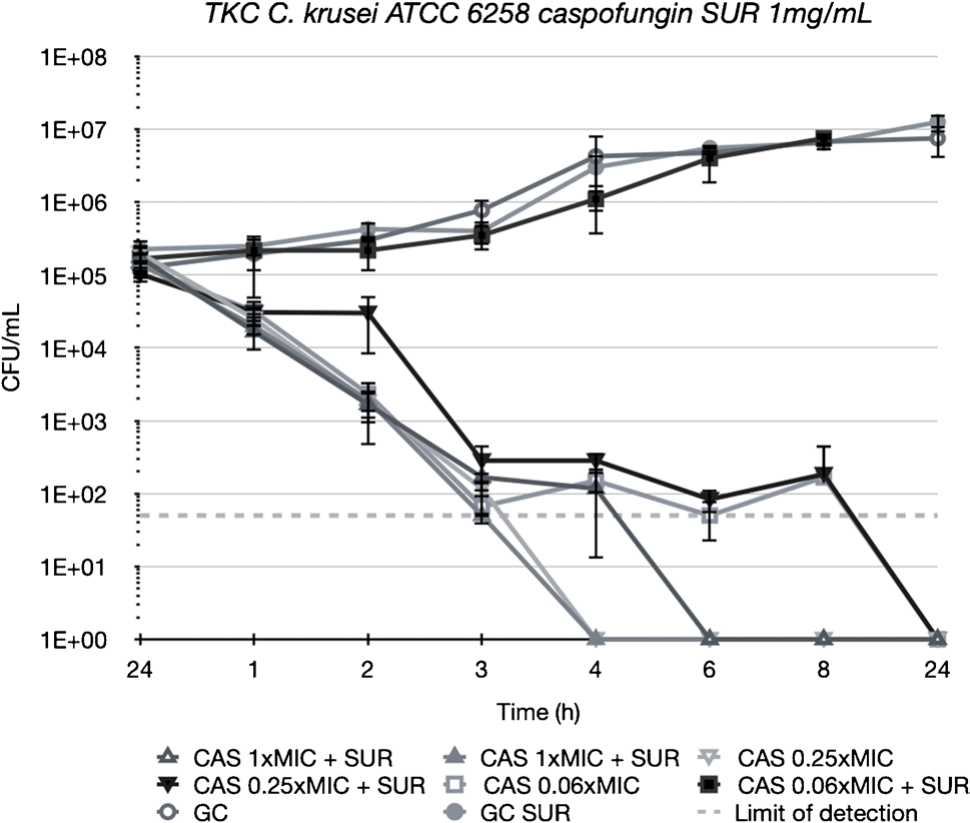
Killing of different concentrations of caspofungin (CAS) against *C. krusei* ATCC 6258 in SDB alone (open symbols) and in the presence of 1 mg/mL of SUR (closed symbols). ANI was tested at 0.06, 0.25 and 1 times its MIC against the test strain

Figures 3, 4, 5 and 6 depict TKCs for *C. krusei* ATCC 6258. In Figs. 3 and 4 (a), TKCs were conducted with ANI and MCF concentrations several times above and equal to the MIC. Additionally, to assess the potential dose-dependent impact of surfactant, different SUR concentrations (0.01, 0.1, and 1 mg/mL) were tested and are presented in Figs. 3 and 4 (b). In both ANI (Fig. 3) and MCF (Fig. 4) TKCs, the addition of SUR completely abolished antifungal activity at a concentration of 1xMIC, resulting in fungal growth curves identical to the growth control. Inhibition of killing was less pronounced at higher antifungal drug concentrations and was either absent or less marked at lower SUR concentrations (0.1 and 0.01 µg/mL).

Similar trends were observed for AMB (Fig. 5), where filled symbols denote concentrations with added SUR, while open symbols represent those without. Complete eradication of *C. krusei* was only attained without SUR presence at concentrations of 1 × and 4 × MIC. In contrast, at identical concentrations with SUR, a reduction of only 1.5 logs and 3 logs was achieved. However, at 0.25 × MIC, no difference in killing was observed after 24 h, regardless of the presence or absence of SUR, despite growth being stalled without SUR for up to 6 h.

No difference in killing was observed with CAS (Fig. 6) when SUR was present, except for at 0.06 × MIC (0.0036 µg/mL), where the surfactant promoted growth until reaching levels equivalent to the growth control.

**Fig. 7:**
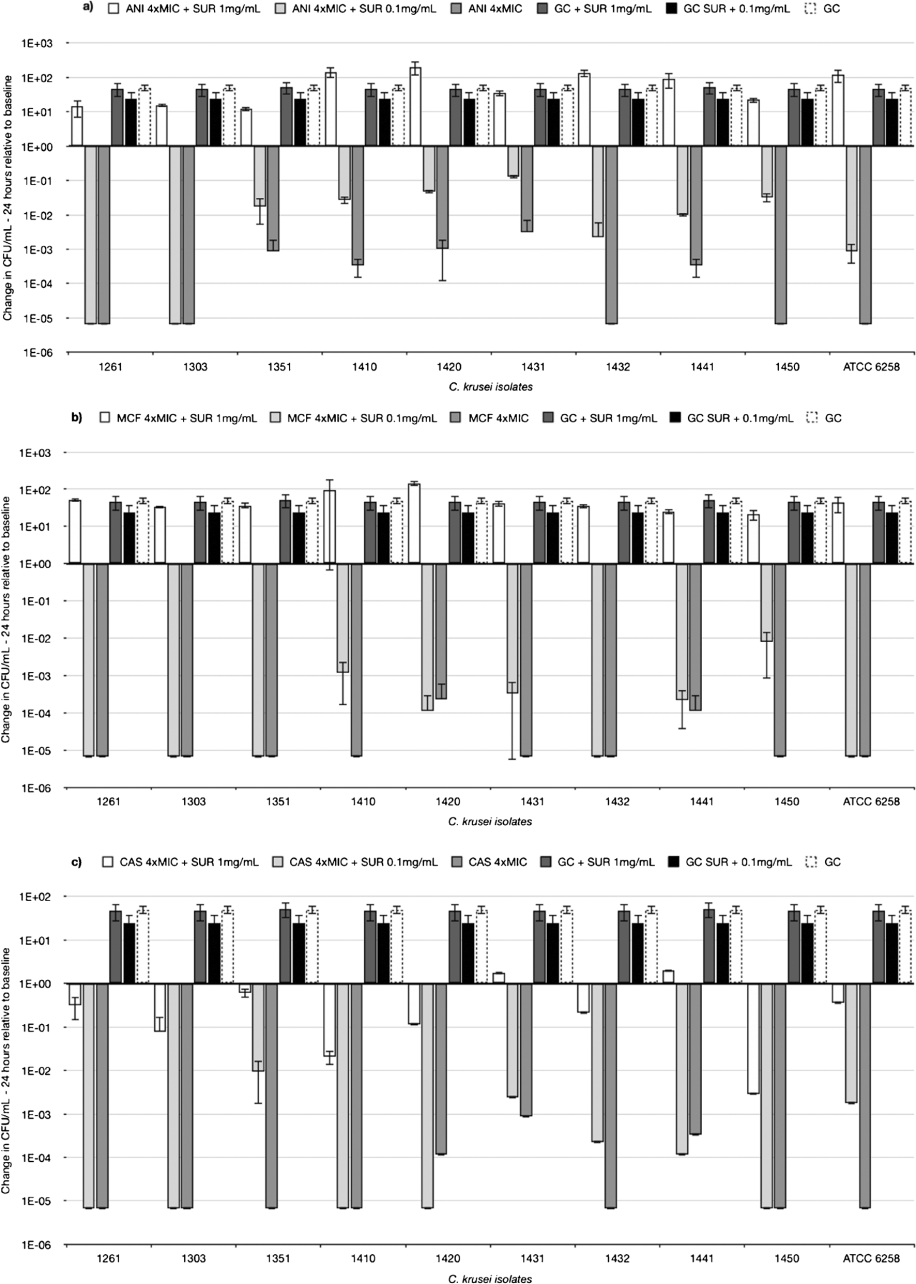
The log10 ratio (CFU/mL at 24 h was divided by the CFU/mL at baseline) indicates the change in CFU/mL over time relative to the baseline. Values greater than 1, indicate growth or an increase in CFU/mL compared to the baseline. Values equal to 0, indicate no change and values less than 1 indicates a decrease or killing in CFU/mL compared to the baseline. Data is shown for clinical isolates of *C. krusei* and ATCC strain 6258 treated with (**a**) anidulafungin, (**b**) micafungin and (**c**) caspofungin*.* All compounds were tested at 4 times their respective MIC against each individual isolate. All compounds were tested alone (white columns) and in the presence of pulmonary surfactant at concentrations of 1 mg/mL and 0.1 mg/mL (filled columns), respectively. GC data is shown for comparison (white column with dashed line)

**Fig. 8:**
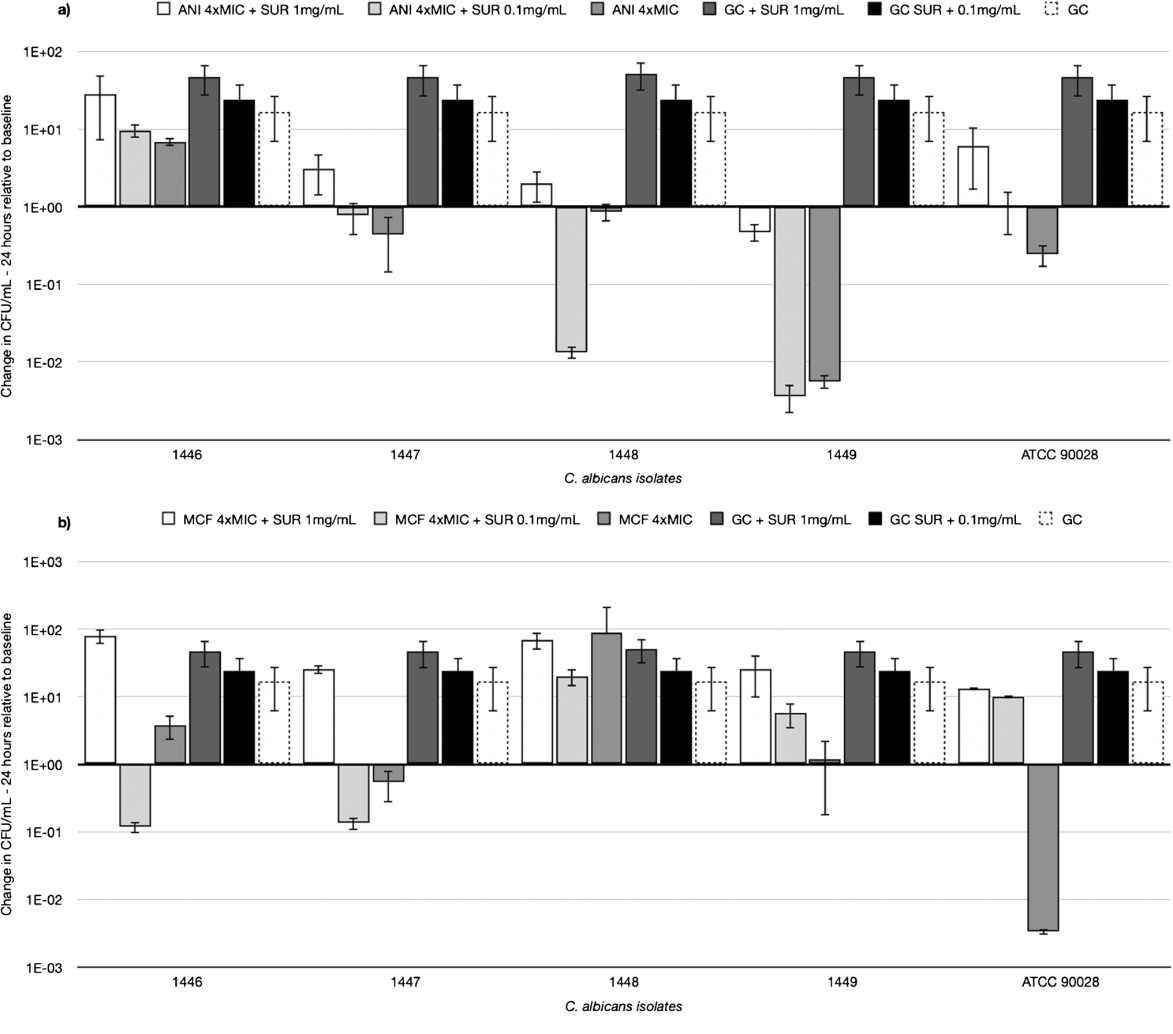
The log10 ratio (CFU/mL at 24 h was divided by the CFU/mL at baseline) indicates the change in CFU/mL over time relative to the baseline. Values greater than 1, indicate growth or an increase in CFU/mL compared to the baseline. Values equal to 0, indicate no change and values less than 1 indicates a decrease or killing in CFU/mL compared to the baseline. Data is shown for clinical isolates of *C. albicans* and ATCC strain 90028 treated with (**a**) anidulafungin and (**b**) micafungin. All compounds were tested at 4 times their respective MIC against each individual isolate. All compounds were tested alone (white columns) and in the presence of pulmonary surfactant at concentrations of 1 mg/mL and 0.1 mg/mL (filled columns), respectively. GC data is shown for comparison (white column with dashed line)

In Mini-TKCS, previous findings were substantiated by experiments using clinical isolates of *C. krusei*, where the addition of SUR reduced the killing of ANI, MCF, and CAS. Figure 7 illustrates the log 10 ratio in CFU/mL (CFU/mL at 24 h divided by the CFU/mL at baseline) indicating the change of CFU/mL over time for each tested isolate at 4xMIC concentrations for ANI (a), MCF (b), and CAS (c), with and without surfactant, alongside GC data for comparison. Concentration-dependent inhibition of antifungal activity by SUR against clinical isolates of *C. albicans* was less pronounced, as depicted by the log10 difference in CFU/mL from 0 to 24 h in Fig. 8 (a) for ANI and (b) for MCF. In contrast to the data observed with *C. krusei*, the effect of surfactant on killing was inconsistent for *C. albicans* ATCC 90028 and the four isolates. Specifically, regarding mini-TKCs with MCF, no killing was achieved for isolate 1446, regardless of the presence or absence of surfactant. Killing of isolates 1447, 1448, and ATCC 90028 was abolished in the presence of 1 mg/mL SUR, while isolate 1449 remained unaffected by any SUR concentration.

Additionally, variable outcomes were observed with ANI. Killing of isolate 1446 was enhanced when 0.1 mg/mL SUR was present, but no killing was observed with 1 mg/mL SUR or without any SUR. Furthermore, killing of isolates 1448 and 1449 was not observed with or without surfactant. The killing of isolate 1447 was impacted by 1 mg/mL SUR, as expected, whereas killing of ATCC 90028 was not influenced by any SUR concentration.

## Discussion

This study aimed to investigate the influence of pulmonary surfactant on the antifungal activity of selected drugs; MCF, CAS, ANI, and AMB against *C. krusei* and *C. albicans*. Whereas considerable knowledge exists on the differential distribution of systemic antifungal agents in the lungs and their association with pulmonary alveolar macrophages and the epithelial lining fluid, little is known about the pharmacodynamic consequences and potential interactions of antifungal agents at these sites [11, 12]. Additive antifungal activity for example against Aspergillus fumigatus has been reported for the combination of amphotericin B formulations with pulmonary alveolar macrophages [13, 14], and that of echinocandins with monocyte-derived macrophages [15, 16]. In addition, in a set of comprehensive in vitro experiments, it was shown that posaconazole (and its parent itraconazole, but not voriconazole) concentrates within cell membranes of pulmonary epithelial cells and rapidly transfers to Aspergillus fumigatus, where it accumulates to high concentrations and persists at the site of its target enzyme to exert antifungal activity [17, 18].

The limited understanding of pulmonary surfactant's impact on antifungal drugs in invasive pulmonary mycoses has prompted exploration, given its critical role in preventing alveolar collapse and its potential as a vehicle for enhanced antibiotic drug delivery, as seen in previous research on daptomycin facing commercial challenges due to inhibited activity in its presence [5, 7, 8].

In the present study, surfactant demonstrated a variable impact on the antifungal activity of the tested drugs. CAS exhibited no significant change in activity, while AMB, MCF, and ANI exhibited a reduction in killing efficacy in the presence of surfactant, ranging from 1 to 5 log steps, against both *C. krusei* (e.g., ATCC 6258, displaying a 5 log step reduction at 1 × MIC of MCF with SUR present at 1 × 10^7 CFU/mL compared to without SUR at 1 × 10^2 CFU/mL) and *C. albicans* (e.g., ATCC 90028, showing a 4 log step reduction at 1 × MIC of ANI with SUR present at 1 × 10^7 CFU/mL compared to without SUR at 1 × 10^3 CFU/mL). The concentration-dependent inhibition of SUR observed in clinical isolates of *C. krusei* (as illustrated in Fig. 7) further underscores the nuanced interaction between surfactant and antifungal drugs, implying that the unique microenvironment of the alveoli could impact the therapeutic effectiveness of antifungal drugs.

In contrast to *C. krusei*, the data regarding the impact of surfactant on *C. albicans* appears to be strain and antifungal specific. As noted by Gil-Alonso et al., *Candida spp.* exhibit varying susceptibilities to echinocandins, even within the same species. Therefore, when antifungal treatment fails, it may be attributed to the fact that different isolates of the same species do not respond equally to antifungals [19].

While our study sheds light on the surfactant-mediated impact on antifungal activity, the specific mechanisms remain elusive. Further investigation is warranted to understand the intricate interactions between pulmonary surfactant and antifungal drugs at a molecular level, offering deeper insights into the observed effects. It's important to note that post-antifungal effects have not been tested specifically in our study, even though this aspect might be of interest, particularly for echinocandins.

As described in a recent publication, surfactants contain high amounts of phospholipids, which could potentially underlie the observed interaction with antimicrobials [6]. Moreover, the origin of surfactant might also influence antifungal activity. Surfactant formulations can vary in their composition, as phospholipids may be sourced from bovine or porcine origins, potentially impacting this interplay [6]. Investigating these factors could provide valuable insights into optimizing antifungal therapy in the context of pulmonary surfactant.

This study provides valuable insights into the variable impact of pulmonary surfactant on antifungal activity. Strengths include the comprehensive assessment of multiple antifungal drugs and the consideration of both standard strains and clinical isolates. In the guideline "Clinical Practice Guideline for the Management of Candidiasis: 2016 Update by the Infectious Diseases Society of America," emphasis is placed on the recognition that invasive infection caused by *Candida spp*. is primarily linked to medical progress, constituting a major contributor to morbidity and mortality in healthcare settings. While there are over 15 *Candida spp.* responsible for human disease, more than 90% of invasive cases are attributed to the 5 most prevalent pathogens: *C. albicans*, *C. glabrata*, *C. tropicalis*, C*. parapsilosis*, and *C. krusei*. Despite their individual differences in virulence potential, antifungal susceptibility, and epidemiology, collectively, infections caused by these organisms are commonly referred to as invasive candidiasis [20]. Moreover, in Erami et al.'s study, colonization with *Candida* was identified in 69 out of 100 immunosuppressed COVID-19 patients, highlighting the prevalence of *Candida spp*. colonization [21]. These studies support our decision to choose *C. albicans* and *C. krusei* isoaltes as relevant test strains in our study.

Moreover, different surfactant concentrations (from 0.01 mg/L to 1 mg/L) have been tested to cover a range of physiological surfactant concentrations achieved in the patient’s lung. However, the limitations lie in the in vitro nature of the study, necessitating further validation in clinical settings, and the need for exploration of potential mechanisms (e.g. molecular investigations) underlying the observed effects. Furthermore, the use of porcine-derived surfactant, Curosurf®, may not entirely capture the characteristics of human surfactant. However, obtaining human surfactant involves invasive lavage procedures, presenting a limitation in the study.

In conclusion, this in vitro study sets the stage for further research to validate and extend these findings in clinical settings. Additionally, this study prompts consideration that assessing antifungals in standard growth media could lead to an overestimation of the agents' efficacy at the intended site of action. Future studies should explore the underlying mechanisms, assess the clinical relevance testing concentrations of antimycotics reached at the target sites, and consider the broader implications for the treatment of invasive pulmonary mycoses.

## Data Availability

Not applicable.
